# Morphological and histological features of abdominal glands in Japanese marten (*Martes melampus*)

**DOI:** 10.1371/journal.pone.0334743

**Published:** 2025-11-07

**Authors:** Jumpei Tomiyasu, Mimi Arakaki, Tetsuhito Kigata, Akio Iwashita, Wataru Tonomori

**Affiliations:** 1 Obihiro University of Agriculture and Veterinary Medicine, Obihiro, Hokkaido, Japan; 2 Tokyo University of Agriculture and Technology, Tokyo, Japan; 3 Tsushima leopard cat Wild Acclimatization Station, Tsushima, Nagasaki, Japan; 4 Department of Geology and Paleontology, National Museum of Nature and Science, Tsukuba, Japan; 5 Ashoro Museum of Paleontology, Ashoro, Japan; Namik Kemal University: Tekirdag Namik Kemal Universitesi, TÜRKIYE

## Abstract

The Japanese marten (*Martes melampus*) is a solitary mustelid species with a strict territorial space that might be maintained by scent marking. However, whether Japanese martens have scent glands that secrete chemical signals remains unknown. We aimed to clarify whether the abdominal glands in these animals secrete chemical signals and, if so, to characterize their morphological and histological features. We investigated nine Japanese martens (eight *M. m. tsuensis* and one *M. m. melampus*) that were all roadkilled. Regardless of sex, season, and subspecies, they all had abdominal glands located at the rostral aspect of the penis or vagina, and brown secretions were evident on the skin area. Enlarged sebaceous and small apocrine glands were spread mostly throughout the abdominal glands. Obviously enlarged, specialized glands were located in the caudal and medial areas of the abdominal glands. The specialized and sebaceous gland cells were connected through a duct at the border between them. This suggested that the specialized gland cells were derived from the sebaceous type. However, eosin staining of the specialized gland cells possessing a nucleus was strongly positive for cytoplasm, whereas that of the sebaceous gland cells was weakly positive. Moreover, the specialized gland cells were weakly stained with Oil Red O, whereas the sebaceous gland cells were strongly stained. Thus, the secretory mechanism of the abdominal specialized glands may not be holocrine like sebaceous glands. In conclusion, Japanese martens had characteristic abdominal glands with developed sebaceous and specialized glands.

## Introduction

Scent is important for intraspecific communication among reproductive, competitive, and parental relationships [1,2]. Scent signals are emitted from feces and urine, as well as apocrine and sebaceous skin glands [1]. Sebaceous and/or apocrine glands in a defined skin area that become species-specifically enlarged and/or specialized are referred to as cutaneous scent glands [3]. These glands secrete pheromones that evoke behavioral and physiological changes in some mammals such as goats [4] and ring-tailed lemurs [5].

Marten species have cutaneous scent glands that play significant roles in marking territories and conveying information about age, sex, and reproductive status [6,7]. Marten species typically have abdominal glands (also called inguinal, ventral and preputial glands) in the abdomen in front of the genitals, as well as anal sac glands, that emit oily secretions with a characteristic marten odor [8–10].

Japanese martens (*Martes melampus*) are solitary within their defined territorial space except during the breeding season [11]. Therefore, they might maintain territories by scent marking like other mustelids [11], but whether they have abdominal glands that secrete scent signals remains unknown. Japanese martens have two subspecies Hondo Marten (*M. m. melampus*) inhabiting Honshu, Shikoku, and Kyushu and Tsushima Marten (*M. m. tsuensis*) inhabiting the Tsushima Islands. The Tsushima marten is classified as a Near Threatened species in Red list of the Ministry of the Environment, Japan [12], because its survival is threatened by habitat loss and roadkill [13]. Understanding how Japanese Martens communicate with others is essential for elucidating intraspecific relationship, which is important for effective conservative efforts. Thus, we aimed to determine whether Japanese martens have abdominal glands, and if so, to characterize their morphological and histological features.

## Materials and Methods

### Animals

For our research, nine martens (eight *M. m. tsuensis* from Tsushima, Nagasaki; one *M. m. melampus* from Hinode, Tokyo, Japan) collected as roadkilled by officers and citizens were frozen at −30°C till the necropsy. Table 1 describes the subspecies, sex, season, date of collection, as well as body weight and length. The time interval from the death to freezing was speculated to be less than 24 hours. The durations of freezing till the necropsy were 512 ~ 1672 days (Average 880 days). We defined the breeding season as July to September [14]. Although pathological findings were absent, we analyzed only samples without severe damage and/or corruption. All experimental procedures complied with relevant laws and the Regulations on Management and Operation of Animal Experiments at Obihiro University of Agriculture and Veterinary Medicine. The Animal Care and Use Committee at Obihiro University of Agriculture and Veterinary Medicine approved the experimental protocol (Approval ID: 24–177). This study proceeded with the approval of Agency for Cultural Affairs because *M.* *m.* *tsuensis* is a designated protected Natural Monument species in Japan.

**Table 1:** Characteristics of nine Japanese martens.

ID	Subspecies	Sex	Breeding season^1^	Collection date^2^ (dd/mm/yy)	Body weight (kg)	Body length (cm)
A	*M. m. tsuensis*	Male	No	19/3/2020	1.6	60.5
B	*M. m. tsuensis*	Male	Yes	18/9/2021	1.2	59.0
C	*M. m. tsuensis*	Female	No	3/12/2021	1.0	56.0
D	*M. m. tsuensis*	Female	No	7/3/2022	1.0	55.0
E	*M. m. tsuensis*	Male	No	16/3/2022	1.4	59.0
F	*M. m. tsuensis*	Female	Yes	28/7/2022	1.1	55.8
G	*M. m. tsuensis*	Female	No	30/11/2022	1.3	55.0
H	*M. m. tsuensis*	Male	No	5/12/2022	1.6	61.0
I	*M. m. melampus*	Male	No	30/7/2024	NA	NA

^1^ Breeding season was defined as July–September as described [Okano and Onuma 2015]. ^2^All collected Martens were roadkilled.

*M. m., Martes melampus*; NA, not analyzed.

### Methods

Skin specimens collected from thawed animals were fixed in 10% formalin, embedded in paraffin using standard procedures, sliced into 5-μm thick sections and histologically stained with hematoxylin-eosin (HE) [15]. Sebum production in skin glands was characterized by staining lipids using Oil Red O [16]. Formalin-fixed tissues were cryoprotected in 0.1 M phosphate buffer containing 20% sucrose overnight and embedded in O.T.C. compound (Sakura Finetek, Tokyo, Japan). Embedded tissues were placed on an aluminum block that had been frozen in liquid nitrogen, then sliced into 10-μm thick sections using a Tissue-Tek Polar cryostat (Sakura Finetek) and mounted on Mas-coated slides. Sliced sections were stained with hematoxylin and Oil Red O (Merck, Darmstadt, Germany) and assessed using an SMZ1500 stereomicroscope and a Microphot-FX microscope (Nikon) equipped with a Digital Sight DS-5M camera (all from Nikon, Tokyo, Japan). Data are presented as means ± standard deviation (SD).

## Results

### Morphological findings of abdominal glands in Japanese martens

**Fig 1:**
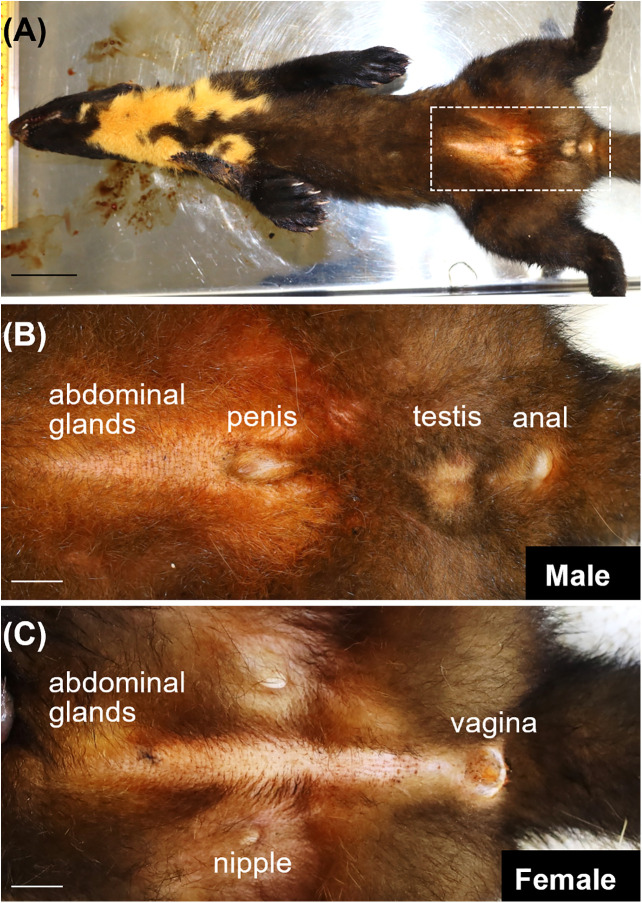
Abdominal glands in Japanese martens. **(A)** Ventral view of Japanese marten. Abdominal glands in rostral aspect of penis in male **(B)** and vagina in female **(C)** martens. Panel **(B)** corresponds to area enclosed by dotted line in panel **(A)**. Scale bars (cm) = 5 **(A)** and 1 **(B, C)**.

Abdominal glands were located at the rostral aspect of the penis or vagina (Fig 1). The secretion on the skin area had a very strong marten odor and the color was brown. The size of abdominal glands was 5.1 ± 1.0 × 0.8 ± 0.3 cm.

### Histological findings of abdominal glands in Japanese marten

**Fig 2:**
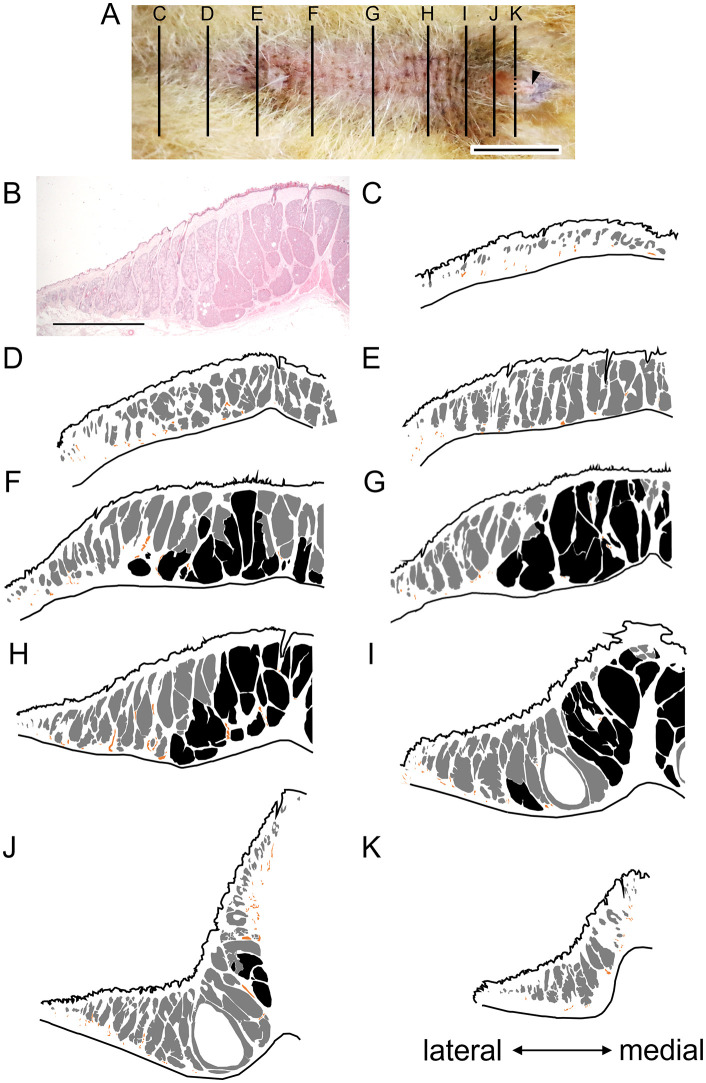
Morphological features of abdominal glands in male Japanese martens. **(A)** Ventral view of abdominal area. Arrowhead, penis. **(B)** Histological image of transverse section of abdominal glands, corresponding to line **(H)** in panel **(A)**. **(C–K)** Sections of sebaceous (grey), specialized (black) and apocrine (orange) glands. Lines in **(A)** correspond to rostral **(C)** and caudal **(K)** positions. Epidermal and dermal sides are respectively up and down. The magnification of panels from **(B)** to **(K)** is the same. Scale bars = 1 cm **(A)**, and 5 mm **(B)**.

**Fig 3:**
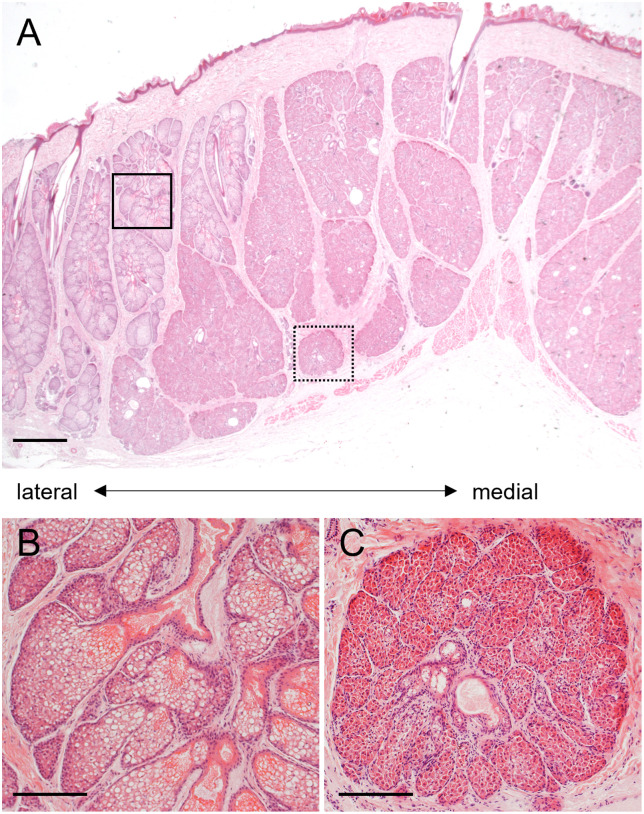
Histological features of abdominal glands in male Japanese marten. **(A)** Transverse-section of abdominal glands. Magnification of sebaceous **(B)** and specialized **(C)** abdominal glands. Solid and dotted squares in panel **(A)** respectively correspond to panels **(B)** and **(C)**. Representative image of caudal areas in abdominal glands, corresponding to panel **(H)** in Figure 2. Scale bars = 1 mm **(A)** and 200 μm **(B, C)**.

**Fig 4:**
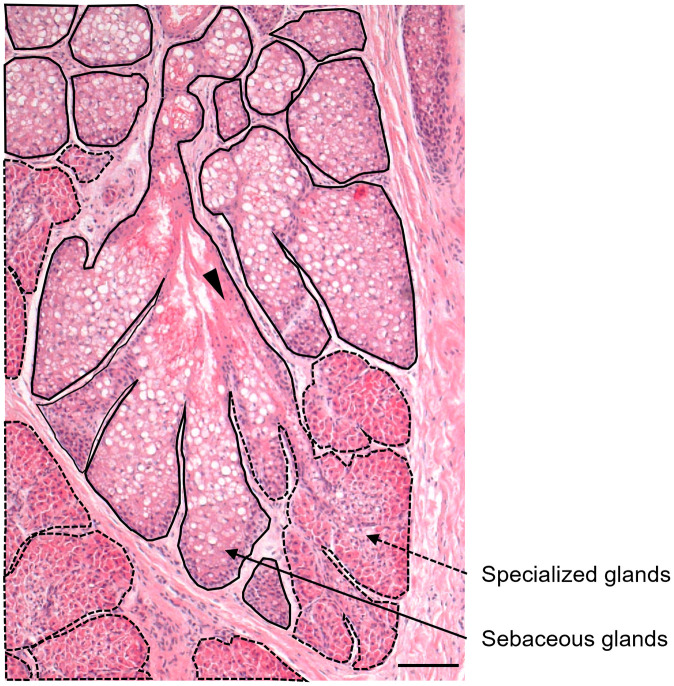
The connection of ducts between sebaceous and specialized glands in the border area. Sebaceous and specialized glands were respectively enclosed by solid and dotted lines. Specialized glands are connected with a duct of sebaceous glands (arrowhead). Scale bar = 100 μm.

**Fig 5:**
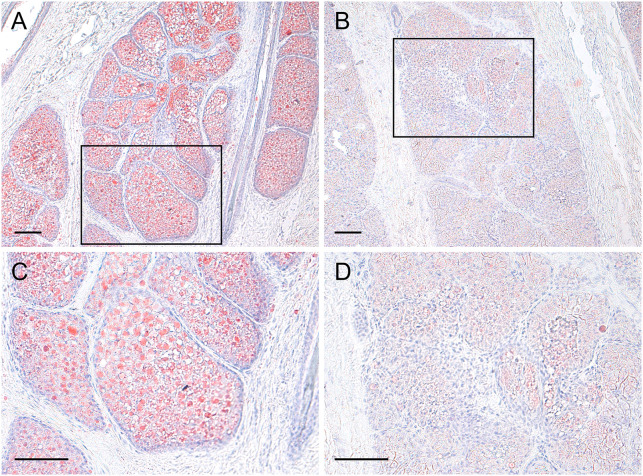
Oil red O stained sebaceous (A,C) and specialized (B,D) abdominal glands in Japanese marten. Squares in panels **(A)** and **(B)** respectively correspond to panels **(C)** and **(D)**. Scale bar = 100 μm (A,B,C,D).

**Fig 6:**
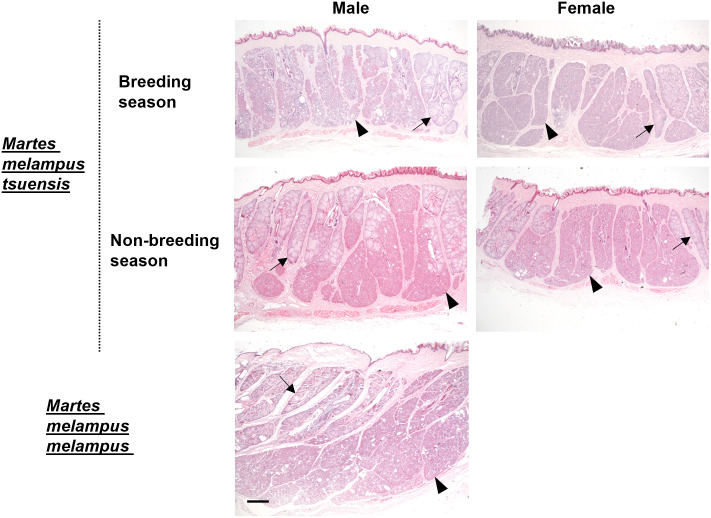
Enlarged sebaceous and specialized abdominal glands of Japanese martens that differed in terms of sex, season and subspecies. Arrow and arrowhead respectively indicate sebaceous and specialized glands. The magnification of the all panels is the same. Scale bar = 1 mm.

Enlarged sebaceous and small apocrine glands were found in most areas of the abdominal glands, (Figs 2, 3, Supplementary data S1 Fig). Enlarged and specialized glands were located in the caudal and medial areas of the abdominal glands (Figs 2, 3, Supplementary data S1 Fig). The distribution of sebaceous and specialized glands among the abdominal glands did not differ between males and females (Fig 2, Supplementary data S1 Fig). The specialized glands were connected with a duct from enlarged sebaceous glands at the border between them (Fig 4). Therefore, the specialized glands might be derived from sebaceous glands. However, the histological features of these glands differed (Figs 3, 5). Cytoplasm in the specialized gland cells possessing nuclei were strongly stained with eosin (Fig 3C). In contrast, the cytoplasm in the sebaceous gland cells was weakly stained with eosin (Fig 3B). The sebaceous gland cells showed strong staining with Oil Red O, whereas the specialized gland cells showed weak staining (Fig 5). The abdominal glands in the nine martens contained enlarged and specialized glands regardless of differences in sex, season, and subspecies (Fig 6).

## Discussion

We found abdominal glands in all nine Japanese martens regardless of physiological status and genetic background. Pine, pacific, American and sable martens, ferrets, American wolverines and stoats have abdominal glands [8,17–20]. Marking territories based on scent secreted from abdominal glands is important for communication [17,21], which should also apply to Japanese martens.

Our histological findings showed that the Japanese marten has abdominal glands with mature sebaceous glands. The abdominal glands of pine and stable martens also include mature sebaceous glands [19,20], whereas those in ferrets contain mainly apocrine glands [18]. Thus, abdominal glands in Japanese martens had a typical feature of the marten species. However, notably, the abdominal glands in Japanese martens had specialized glands in the specific area, the caudal and medial area. Abdominal glands in pine martens are exclusively sebaceous and do not differ regionally [19]. Therefore, the specialized glands are characteristic in Japanese martens.

Because they are connected, we consider that the specialized glands are derived from sebaceous glands. The secretory mechanism of sebaceous glands is holocrine secretion. The basal layer contains cells that actively undergo mitosis. As they migrate towards the gland duct from the basal layer, they become enlarged, denucleated, and degenerate, thus releasing their contents, including stored sebum. [3]. The specialized gland cells in the Japanese martens did not become enucleated, and were stained weakly positive for lipids. In contrast, the sebaceous gland cells were stained strongly for lipid, suggesting that the specialized glands are not holocrine. Understanding how the specialized glands in the abdomens of Japanese martens secrete chemicals will require ultrastructural analysis of fixed fresh tissues.

In our results, Japanese marten had specialized sebaceous glands in the abdominal skins. Chemical signals are produced and secreted from cutaneous skin glands, the apocrine and sebaceous glands [1], and both skin glands generally differ in the properties and functions of the secretions [1,21]. The secretions of apocrine glands, which contain hydrophilic molecules, typically volatilize fast as alarm pheromones. An example is the metatarsal glands of black-tailed deer [22,23]. In contrast, the secretion of sebaceous glands, including lipids, volatilizes relatively slowly [1]. Because the smell retains for a long time, it may work for the marking [1]. Examples are perineal glands in hamsters [24] and midventral glands in Mongolian Gerbil [25]. Considering the general function of sebaceous glands secretions, the secretion of abdominal glands in Japanese Marten may be important for the marking behaviour for territorial maintenance.

Scent glands can actively secrete during a specific season or all seasons. Seasonal and sexual variations in secretory activity are obvious in the back scent glands of brown bears [26], forehead glands in impalas [27] and the pre-orbital glands of reindeer [28]. These secretory activities correlate with reproductive cycles, implying these glands play significant roles during the breeding season. In contrast, secretory activity in males and females does not change seasonally in the metatarsal glands of impalas [28] and in the caudal and tarsal glands of reindeer [29]. This suggests a general social role for communication with other individuals. We revealed that the abdominal glands of Japanese martens could secrete scent during any season. Abdominal marking is important for territorial ownership among stoats in agonistic situations [17]. Male and female stoats rub abdominal glands secretions at sites where they capture prey and replace other stoats throughout the year [17]. Japanese martens have very strict territory [11] that might be defined by abdominal gland secretions.

In conclusion, we uncovered the morphological and histological features of the abdominal glands of Japanese martens. These glands are mostly sebaceous and, specialized glands are located in caudal and medial areas.

## Supporting information

S1 Fig Morphological features of abdominal glands in female Japanese martens. (A) Ventral view of abdominal area. Arrowhead, vagina. (B-H) transverse sections of sebaceous (grey), specialized (black) and apocrine (orange) glands in abdominal glands. Dotted lines in (A) correspond to rostral (B) and caudal (H) positions. Epidermal and dermal sides are respectively up and down. The magnification of the panels from (B) to (H) is the same.
(PDF)
