# Supplementary material for: Morphological and histological features of abdominal glands in Japanese marten (Martes melampus)
Source: PLoS One. 2025 Nov 7;20(11):e0334743. doi: 10.1371/journal.pone.0334743 (PMC12594411; doi:10.1371/journal.pone.0334743)
Supplement: S1 Fig — (A) Ventral view of abdominal area. Arrowhead, vagina. (B-H) transverse sections of sebaceous (grey), specialized (black) and apocrine (orange) glands in abdominal glands. Dotted lines in (A) correspond to rostral (B) and caudal (H) positions. Epidermal and dermal sides are respectively up and down. The magnification of the panels from (B) to (H) is the same. (PDF) [file pone.0334743.s001.pdf]

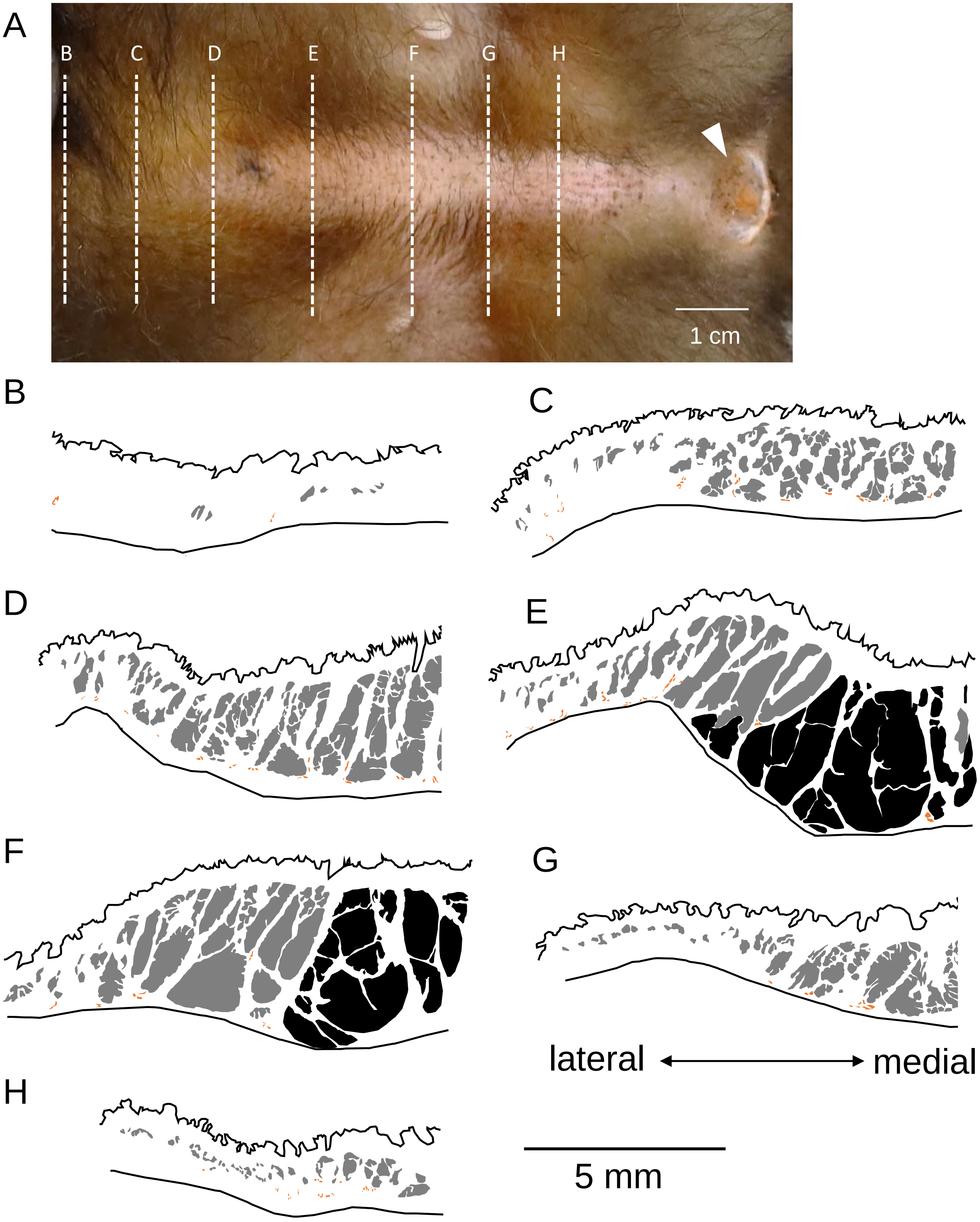

Supporting information. Morphological features of abdominal glands in female Japanese martens. (A) Ventral view of abdominal area. Arrowhead, vagina. (B-H) transverse sections of sebaceous (grey), specialized (black) and apocrine (orange) glands in abdominal glands. Dotted lines in (A) correspond to rostral (B) and caudal (H) positions. Epidermal and dermal sides are respectively up and down. The magnification of the panels from (B) to (H) is the same.
